# Effects of Dietary Supplementation Using Phytobiotics with Different Functional Properties on Expression of Immunity Genes, Intestinal Histology, Growth, and Meat Productivity of Broiler Chickens

**DOI:** 10.3390/vetsci12040302

**Published:** 2025-03-25

**Authors:** Marina I. Selionova, Vladimir I. Trukhachev, Artem Yu. Zagarin, Egor I. Kulikov, Nina P. Belyaeva

**Affiliations:** 1 Department of Animal Breeding, Genetics and Biotechnology, Institute of Animal Science and Biology, Russian State Agrarian University—Moscow Timiryazev Agricultural Academy, 49 Timiryazevskaya Str., Moscow 127434, Russia; selionova@rgau-msha.ru; 2 Department of Animal Nutrition, Institute of Animal Science and Biology, Russian State Agrarian University—Moscow Timiryazev Agricultural Academy, 49 Timiryazevskaya Str., Moscow 127434, Russia; rector@rgau-msha.ru; 3 Laboratory of Applied Genetics, Federal State Budget Scientific Institution Federal Scientific Center “All-Russian Research and Technological Poultry Institute”, 10 Ptitsegradskaya St., Sergiev Posad, Moscow 141311, Russia; kulikovegor33@yandex.ru; 4 Department of Morphology and Veterinary Sanitary Examination, Institute of Animal Science and Biology, Russian State Agrarian University—Moscow Timiryazev Agricultural Academy, 49 Timiryazevskaya Str., Moscow 127434, Russia; anatomy_muz@rgau-msha.ru

**Keywords:** animal nutrigenomics, crypt depth, defensins, interleukin, intestinal health, plant extracts

## Abstract

The aim of this study was to investigate the expression of immunity-related genes and morpho-histological features of the intestines, and the growth and meat production of broiler chickens when fed plant extracts with different functional components. Chickens in the control group received a basic diet. The feed in the experimental groups contained plant extracts standardized to various biologically active components such as the extract of common chicory with inulin (INUL), St. John’s wort with flavonoids (FLAV), maral root with ecdysterone (ECDS), and extracts of creeping thyme with flavonoids and tannins (FLAV-TANN). The results of this study showed that the application of the studied phytobiotics increased the expression of the pro-inflammatory gene *IL8*, with the 2^−ΔΔCT^ value ranging from 2.66 to 4.63. In the case of the antimicrobial peptide gene *AvBD9*, the 2^−ΔΔCT^ value ranged from 1.66 to 8.16, depending on the group. *AvBD10* gene expression increased (2^−ΔΔCT^ = 2.19) when the chickens were fed the chicory extract and decreased when thyme extract was used (2^−ΔΔCT^ = 0.33). The study also found that using biologically active components in the diets of broiler chickens was accompanied by a significant decrease in the height of epithelial cells in the cecum mucous membrane in the INUL group (61.3%, *p* ≤ 0.001), FLAV group (60.0%, *p* ≤ 0.001), ECDS group (48.2%, *p* ≤ 0.001), and FLAV + TANN group (67.6%, *p* ≤ 0.001). It also caused a reduction in the depth of crypts in the INUL (38.4%, *p* ≤ 0.001), FLAV (32.3%, *p* ≤ 0.001), ECDS (50.9%, *p* ≤ 0.001), and FLAV + TANN (53.4%, *p* ≤ 0.001) groups. The use of all extracts, except creeping thyme, caused changes in the size of muscular elements in the intestinal walls; thus, the thickness of the muscular layer increased 1.5–2.0 times under the influence of flavonoids in St. John’s wort and of inulin in chicory, to 430.99 and 579.87 μm, respectively (*p* ≤ 0.001), and decreased 1.9 times under the influence of ecdysterone in maral root to 151.59 μm (*p* ≤ 0.001). The use of phytobiotics increased feed consumption and stock safety, leading to an increase of 4.1–7.5% in the live weights of broiler chickens at the end of the rearing period, thus contributing to the higher slaughter quality of poultry; in particular, the weights of breast muscles in cockerels in the INUL group increased by 16.9% (*p* ≤ 0.05), while the weights in the ECDS and FLAV + TANN groups increased by 18.1% (*p* ≤ 0.05) and 23.1% (*p* ≤ 0.01), respectively. Thus, the use of the studied phytobiotics in the broiler chicken diet increases meat production and activates immunity, which indicates the possibility of replacing antibacterial drugs with natural nutraceuticals.

## Simple Summary

One of the key areas of healthy nutrition in monogastric animals is the replacement of feed antibiotics with environmentally safe alternative nutraceuticals, in particular, phytobiotics, which, among other beneficial properties, can have a beneficial effect on intestinal health. The key biomarkers that can be used to determine the effect of diet on intestinal health can be identified by analyzing the transcriptional activity of genes associated with antimicrobial and antiviral defense, inflammation initiation, and morpho-histological parameters of the intestinal epithelium. his study considered the effects of the phytobiotics of plant extracts with different biologically active components on productivity in poultry and their health status. The results of the study showed the advantage of thyme extract over other phytobiotic extracts such as common chicory, St. John’s wort, and maral root.

## 1. Introduction

The antibiotic resistance of pathogenic microorganisms resulting from the uncontrolled use of feed antibiotics as growth stimulants in livestock production is becoming one of the most urgent problems in veterinary and public health [1,2]. Resistance to antibacterial agents circulates through the environment, from animals to humans. The key link in this chain is agricultural animals, especially poultry, for which antibiotics are widely used to prevent and treat infectious diseases and to stimulate growth. Antibiotic-resistant bacteria can be transmitted from animals to humans through the consumption of contaminated food products and the slaughter and processing of carcasses, as well as indirectly through animal waste that pollutes the environment [3].

The global trend to reduce the use of antibiotics in livestock production has stimulated the development of research on alternative feed additives that act as natural growth stimulants, including prebiotics, probiotics, organic acids, enzymes, and phytobiotics. Phytobiotics with pharmacological properties have been used in alternative medicine due to their antibacterial and immunomodulatory effects, which justifies their use as animal feed additives [4]. Phytobiotics are obtained from herbs, spices, extracts, and essential oils containing a wide range of biologically active components, depending on the botanical composition, origin, and processing method [5].

Phytobiotics have a positive effect on the productivity and health of farm animals. Previous studies have revealed the useful properties of phytobiotics such as an increase in productivity, feed consumption, gene expression level, the intensification of metabolism and the increased efficiency of feed utilization, the improvement of the morphofunctional properties of the intestine, and the stimulation of digestive enzyme secretion [6].

An important function of phytobiotics is immunity modulation. In particular, phytobiotics contribute to the increase in intestinal barrier function and antibacterial and antiviral defense in animals [7]. Multiple studies indicate a positive effect of phytocomponents on the proliferation of the total number of leukocytes, as well as T- and B-lymphocytes in blood [8], changes in the expression of inflammation genes in the intestines [9], an increase in antibody titers, and other positive changes in innate immunity [10].

The use of modern molecular genetic methods enables the objective assessment of the influence of the active components in nutraceuticals on the physiological status of animals and the processes that underlie the formation of productivity traits and the standard of animal well-being. Studies of target genes expression associated with a specific function reveal the influence of analyzed factors, including nutrition, on specific changes in animal organisms, and establish whether a feed product exhibits biologically active effects. Studies based on this methodology have generated a new scientific discipline—nutrigenomics in animal rearing [11]. Thus, nutrigenomic analysis is used to identify the effect of phytobiotics on the expression of genes related to the growth and meat productivity of broiler chickens [12], nutrient transport and antioxidant defense [13], and the activity of digestive enzymes, as well as lipogenesis, immunity, and autophagy [14]. However, there are currently insufficient data on the comparative analysis results regarding biologically active compounds of plant origin in the literature, especially in terms of the effects of different functional properties on the expression of genes related to the intestinal health of broiler chickens and comparisons of molecular data with histological markers; thus, our study is novel in this regard.

This study, formulated on the basis of a literature review, examines the possibility of improving the productivity and intestinal health of broiler chickens through the use of plant extracts in the diet. Specifically, the aim was to study the expression of immunity-related genes and histological parameters in the ceca tissue, growth patterns, and the meat productivity of broiler chickens fed with plant extracts with different biologically active components.

## 2. Materials and Methods

The animal study was reviewed and approved by the Bioethics Commission of the Russian State Agrarian University—Moscow Timiryazev Agricultural Academy (Protocol No. 16, 30 January 2024).

### 2.1. Animals and Experimental Design

The experiment was carried out in accordance with the recommendation of the Board of the Eurasian Economic Commission, 14 November 2023, No. 33, “On methodological recommendations for working with laboratory (experimental) animals in preclinical (non-clinical) studies”. This study was conducted on Russian crossbred ‘Smena 9’ broiler chickens from 22 March to 26 April 2024 in the training and production poultry house (vivarium) of the Russian State Agrarian University—Moscow Agricultural Academy, named after K.A. Timiryazev (Moscow, Russia). After the completion of incubation, conducted at the Centre of Genetics and Selection ‘Zagorskoye EPH’ (Sergiev Posad, Russia), 180 chickens were divided into 5 groups and 3 replications with 36 animals in each group and 13 individuals in each replication (Table 1) using the analog balanced group method and considering live weight and overall development. Hens and cockerels were randomly allocated.

The first group of broiler chickens was the control group and, throughout the entire rearing period, received complete compound feed without the inclusion of biologically active components. In the experimental groups, different plant extracts were introduced into the composition of compound feeds (“Starter”/“Grower”/“Finisher”), which were standardized for different biologically active components. In experimental group 2, common chicory extract (*Cichorium intybus* L.) was introduced into the feed at 450–560 g/t, which was equivalent to 405.0–504.0 g of inulin (INUL) per ton of feed. In experimental group 3, we used 350–430 g/t extract of St. John’s wort shoots (*Hypericum perforatum* L.), which was equivalent to 22.6–31.4 g/t of flavonoids (FLAV). In experimental group 4, we added 170–210 g/t of maral root extract (*Rhaponticum carthamoides* L.), which was equivalent to 0.9–1.1 g/t of ecdysterone (ECDS). In experimental group 5, we used 200–250 g/t of thyme shoot extract (*Thymus serpyllum* L.), which was equivalent to 36.8–46.0 g/t of flavonoids and 7.20–9.00 g/t of tannins (FLAV + TANN), respectively. The amount of plant extract to be added into the compound feeds was selected for the first time. However, when selecting the levels of phytobiotic additions for the mixed fodders, the authors took into account the results of previous studies, thus confirming positive effects observed as a result of similar dosages of common chicory [15], St. John’s wort [16], maral root [17], and thyme [18].

All chicks were kept in the same area in cages with a wire floor, strictly following the technological parameters, e.g., temperature, humidity, planting density, and lighting, approved by the developers of the crossbreed [19].

The room temperature was maintained at

- 31–33 °C—in the first week;
- 24–30 °C—in the second and third weeks;
- 20–23 °C—in the fourth and fifth (final) week of the experiment.

Relative air humidity varied from 40 to 60% in the first week to 60–70% throughout the experiment.

The light schedule during the broiler chick rearing period was maintained using an automatic timer:

- On the last day of incubation when the chicks hatched—constant light;
- Days 1–7—23 h of light and 1 h of darkness;
- Days 8–34—four cycles of 5 h of light and 1 h of darkness;
- Day 35—23 h of light and 1 h of darkness.

The cages were organized in three tiers, with each section measuring 65 × 70 cm. The chicks were placed so that, at the end of the experiment, the number of individuals per unit area was 476 cm^2^ per head, corresponding to 21 chicks per square meter. Each group of chicks was evenly distributed on all levels.

Feed and drink were available ad libitum. Bunker feeders were used for the first 10 days and were then switched to trough feeders from day 11 to 35. Feeding was carried out manually every day.

Drink was available through vacuum drinkers for the first 10 days, after which nipple drinkers were introduced until the end of the experiment. Paper bedding was used for the first 10 days and was subsequently switched to wire flooring.

Thus, the housing and feeding conditions of the chickens in all groups were the same and met the requirements for the crossbreed ‘Smena 9’. The experiment covered the entire broiler chicken growing period and totaled 35 days. It was thus possible to obtain reliable results and evaluate the influence of the studied factors on the growth and development of chickens in the same conditions.

### 2.2. Broiler Chicken Nutrition and Plant Extracts

From the first day of the experiment until day 10, we used a complete compound feed, “Starter”, in the form of pellets; from days 11 to 22, we used a “Grower” feed, in the form of pellets; and from days 23 to 35, we used a “Finisher” feed, in the form of coarse meal. The producer of mixed fodders was JSC ‘Melkombinat’ (Russia). The production of the feed corresponded to the standards set by the company. Guar powder was used as a thickener. The “Starter” contained wheat, soya meal, corn, full-fat soya, soya cake, sunflower cake, meat and bone meal, corn gluten, alcohol yeast, defluorinated phosphate, limestone meal, sunflower oil, lysine sulfate, methionine, choline, threonine, lysine, sodium sulfate, table salt, premix, a synthetic antioxidant, a mycotoxin adsorbent, and an enzyme complex. The “Grower” contained such components as wheat, full-fat soya, corn, sunflower cake, corn gluten, meat and bone meal, sunflower meal, defluorinated phosphate, sunflower oil, lysine sulfate, lysine, methionine, threonine, choline, sodium sulfate, valine, baking soda, premix, a synthetic antioxidant, a mycotoxin adsorbent, and an enzyme complex. The “Finisher” contained the following components: wheat, corn, full-fat soya, sunflower cake, sunflower meal, meat and bone meal, sunflower oil, defluorinated phosphate, lysine sulfate, lysine, methionine, threonine, choline, valine, baking soda, sodium sulfate, table salt, premix, a synthetic antioxidant, a mycotoxin adsorbent, and an enzyme complex. The mixed fodder had a chemical composition, metabolic energy, and amino acid ratio which fully met the recommended standards for feeding the crossbreed ‘Smena 9’ broiler chickens [19] (Table 2).

The dry aqueous extracts of plants were obtained in the form of fine powders of varying shades, namely white (*C. intubus* L.), light brown (*H. perforatum* L., *T. serpyllum* L.), and dark brown (*R. carthamoides* L.); these were used as biologically active components in the experiment. The extracts were obtained from the root systems of chicory and maral root and the shoots of St. John’s wort and thyme. The extracts were produced by two biotechnological enterprises, namely Naturing LLC (Moscow, Russia) (*C. intubus* L. extract), in accordance with ISO 9001:2015 and 22000:2018, and Wisterra LLC (Altai region, Russia) (*H. perforatum* L., *R. carthamoides* L., *T. serpyllum* L. extracts), in accordance with ISO 22000:2005. These specific extracts were selected due to the different functional properties of their key components: growth stimulation (ECDS); the stabilization of intestinal microbiota (INUL); and antimicrobial, antioxidant, and immunomodulatory activity (FLAV, FLAV + TANN). The addition of the extracts to the feed occurred in stages. First, the entirety of the extract was thoroughly mixed with 100 g of mixed fodder in accordance with the experimental design. This mixture was then combined with 900 g of conventional feed to create 1 kg of mixture (100 g with extract + 900 g of conventional). After that, the 1 kg of mixed feed with the extract was mixed together with 9 kg of regular mixed feed, resulting in 10 kg of mixture. The final step was to mix 10 kg of the mixed feed with 40 kg of the conventional feed, yielding 50 kg of finished feed, thus ensuring that the extract was evenly distributed.

### 2.3. Growth and Meat Productivity of Broiler Chickens

To assess the zootechnical efficiency of broiler chicken rearing, the individual weighing of all experimental chickens was conducted on a weekly basis, and the average live weight and average daily gain in each group were subsequently calculated. Livability and feed consumption records were kept in order to correctly calculate feed costs per 1 kg of gain.

Individual weighing was performed on electronic laboratory scales, Mercury 122ACFJR-600.01 (Mertech, Shchyolkovo, Russia), with an accuracy of 0.01 g, and on electronic optical scales, M-ER 223 AC-15.2 Mary LCD (Mertech, Shchyolkovo, Russia), with an accuracy of 2 g. Data on live weight (g) were recorded on special forms for individual weighing.

The average daily gain (ADG, g) was calculated from the weighing results using the following formula:

(1) $$\mathrm{ADG}=\frac{W_{2}-W_{1}}{t_{2}-t_{1}}$$

where W_2_ represents the live weight of broilers at the end of the rearing period in grams; W_1_ represents the live weight of broilers at the beginning of the rearing period in grams; t_2_ represents the age of chicks at the end of the rearing period in days; and t_1_ represents the age of chicks at the beginning of the rearing period in days.

The feed cost per 1 kg of gain (FC) was calculated based on feed consumption records and the gross live weight gain of chickens in each group using the following formula:

(2) $$\mathrm{FC}=\frac{individual\;feed\;intake\;during\;the\;rearing\;period,\;g\;}{average\;absolute\;growth\;of\;chicks,\;g}$$

where the individual feed intake during the rearing period was obtained by dividing the gross feed intake of the group by the number of chicks and taking into account the changes in the birds’ livability during the experiment.

The livability of the poultry was calculated as the ratio of the number of chickens at the end of the rearing period to the number of chickens at the beginning of the rearing period, expressed in percent in accordance with the records.

Based on the results of the experiment, the European Production Efficiency Factor (EPEF) was calculated using the following formula:

(3) $$\mathrm{EPEF}=\frac{livability,\%\times live\;weight\;of\;1\;chicken\;in\;kg}{slaughter\;age\;in\;days\times FC\;for\;1\;kg\;of\;gain\;in\;kg}\times 100$$

After a preliminary 10 h period of fasting to ensure that the gastrointestinal tract was empty, 6 chickens (3 males and 3 females) aged 35 days and with an average live weight according to their sex (*n* = 30: 15 males + 15 females) were selected from each group. The birds were slaughtered and anatomically dissected in accordance with generally accepted methods. The conditions for euthanasia were in accordance with the recommendations of the Board of the Eurasian Economic Commission, 14 November 2023, i.e., No. 33, which comprises methodological guidelines for the treatment of laboratory (experimental) animals during preclinical (non-clinical) research processes.

The anatomical cutting of the carcasses was carried out in a specially prepared room using cutting tools. The results of analyses were recorded in protocols. Internal organs, muscles, and bones were weighed on a laboratory scale, Mercury 122ACFJR-600.01 (Mertech, Shchyolkovo, Russia), with an accuracy of 0.01 g. The following parameters were taken into account during the anatomical cutting of broiler chickens: gutted carcass weight (g), slaughter yield (%), breast muscle weight (g) and its yield (%), and fat weight (g) and its yield (%).

### 2.4. Sample Collection

At 26 days of age, 4 broilers with average live weights were selected from each group. The decision regarding the specific number of individuals needed to select tissues for gene expression analysis was guided by the methodology of a previous study [20]. In accordance with the recommendations of the Board of the Eurasian Economic Commission, 14 November 2023, No. 33, “On methodological recommendations for working with laboratory (experimental) animals in preclinical studies”, the chickens were euthanized and then dissected, and the ceca tissue samples were collected under sterile conditions. The collected samples were stabilized using IntactRNA fixative (Evrogen, Moscow, Russia) and stored at −20 °C until laboratory analysis.

At 32 days of age, 3 chickens from each group with average live weights were selected in a similar manner. Compared to the animals used for gene transcriptional activity analysis, the number of chicks from which tissues were taken for histological studies was reduced due to the subsequent measurement of the histostructure of each sample in 11 replicates. A morphological evaluation of their digestive organs was carried out, and 1 cm^3^ samples of ceca were taken within 15 min of slaughter for histological study at the educational and scientific laboratory of the Department of Morphology and Veterinary Sanitary Examination of the Russian State Agrarian University—Moscow Timiryazev Agricultural Academy. To avoid cell disintegration and the necrosis of intestinal tissues, samples were fixed within 2 min of collection using 10% formaldehyde solution.

### 2.5. RNA Isolation and Real-Time PCR

The gene transcriptional activity was studied in the laboratory of applied genetics of the Federal Research Centre ‘All-Russian Research and Technological Institute of Poultry Breeding’ located in Sergiev Posad, Russia. As before, tissue homogenization and lysis were carried out using the Bioprep-24 instrument (Allsheng, Hangzhou, China). Reverse transcription was performed using the Magnus kit (Evrogen, Moscow, Russia) and GeneExplorer GE-96G thermocycler (Bioer, Hangzhou, China). A Fluo-200 fluorimeter (Allsheng, Hangzhou, China) and a QuDu ssDNA kit (Thermo Fisher Scientific, Waltham, MA, USA) were used to analyze the quality of the extracted RNA and cDNA. All RNA isolation and cDNA synthesis procedures were performed in accordance with the protocols provided by the molecular genetic kit manufacturers. The obtained cDNA was stored at −30 °C.

For the amplification of the selected genes, genome-specific primer sequences were used (Table 3). The primers were selected based on the results of a review examining the global scientific literature and were verified using the Ensembl genome browser. The amplification reaction was performed on a QuantStudio 5 instrument (Thermo Fisher Scientific, Waltham, MA, USA). As a reference gene, we used the “housekeeping” gene encoding the protein β-actin ACTB. Amplification was performed using the 5X qPCRmix-HS SYBR kit (Evrogen, Moscow, Russia) according to the manufacturer’s recommendations: 3 min at 95 °C (preliminary denaturation); 30 s at 95 °C, 30 s at 60 °C, and 30 s at 72 °C (40 cycles). The assessment of the relative expression level was performed using the 2^−ΔΔCT^ method [21].

### 2.6. Histological Studies of Blind Intestinal Pouches

Histological preparations were carried out in triplicate using standard methods, involving the dehydration and substitution of formaldehyde with paraffin and passing it through ethyl alcohol, followed by staining with hematoxylin–eosin. The analyses were performed on a Mikmed-5 microscope (Lomo Ltd., St. Petersburg, Russia) under magnifications ranging from 600× to 1500×. Using an ocular ruler pre-calibrated with an ocular micrometer, 31 measurements of the epithelium height, the crypt depth, the thickness of muscularis mucosae, the submucosa, and the muscular layer were taken for each specimen.

### 2.7. Statistical Analysis

The statistical processing of the data was performed using the IBM SPSS Statistics 23 (2023) program. A one-way analysis of variance (ANOVA) with Tukey’s test was used to analyze growth parameters, anatomical dissection, blood biochemical analysis, and relative gene expression in the experimental groups. The normalized 2^−ΔΔCT^ value was used to calculate relative gene expression. Student’s *t*-test for ΔCt values was used to compare gene expression levels in the experimental groups and the control group. The difference was considered statistically significant at *p* ≤ 0.05.

## 3. Results

### 3.1. Individual Consumption of Phytochemicals by Chickens

The individual consumption of biologically active components by poultry during the entire rearing period was as follows: inulin in *C. intubus* L.—1628 mg in 1.8 g of extract (INUL); flavonoids in *H. perforatum* L.—97 mg in 1.4 g of extract (FLAV); ecdysterone in *R. carthamoides* L.—4 mg in 0.7 g of extract (ECDS); and flavonoids and tannins in *T. serpyllum* L.—29 and 146 mg in 0.8 g of extract (FLAV + TANN).

### 3.2. Expression of Immunity Genes in the Cecum of Broiler Chickens

**Figure 1:**
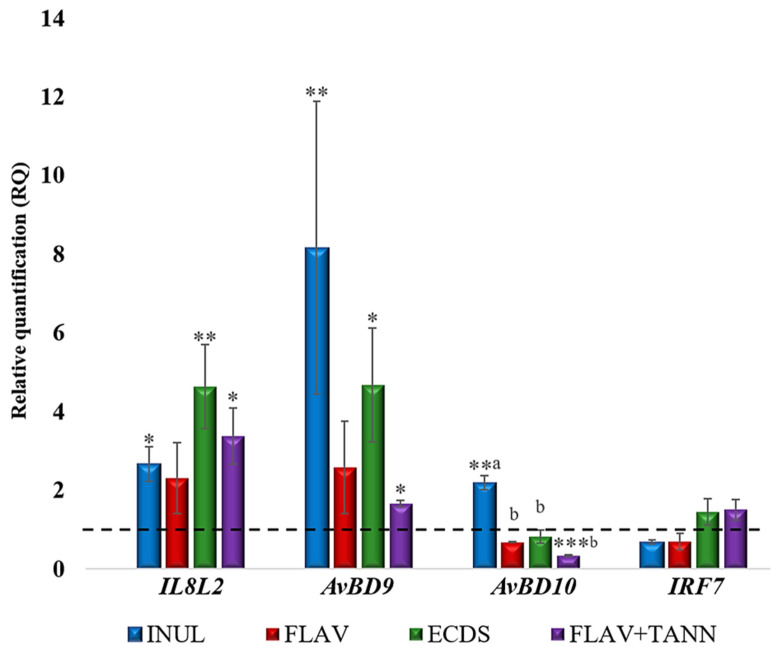
Relative quantification (RQ) of expression of immunity-related genes in the ceca of broiler chickens (the line shows the basal expression level in the control group, taken as 1; results are presented as the means and the standard errors of the means (M ± SEM) for mRNA expression; *, **, and *** represent the differences in ΔCt values that are statistically significant in relation to the control group at *p* ≤ 0.05, *p* ≤ 0.01, and *p* ≤ 0.001, according to the t-criterion; a, b—differences between indices without a common letter are statistically significant at *p* ≤ 0.05; CON—control; INUL—inulin in common chicory (*C. intubus* L.); FLAV —flavonoids in St. John’s wort (*H. perforatum* L.); ECDS—ecdysterone in maral root (*R. carthamoides* L.); FLAV + TANN—flavonoids and tannins in creeping thyme (*T. serpyllum* L.).

The results of the molecular genetic analysis which aimed to identify changes in the expression of immunity-associated genes in the cecum tissues of broiler chickens fed biologically active components extracted from raw plant material are presented in Figure 1.

The results of the gene expression analysis revealed statistically significant increases in the expression of interleukin-8 when different extracts were added to the diet of broiler chickens: inulin (*C. intubus* L.)—increase of 2.66 (*p* ≤ 0.05); ecdysterone (*R. carthamoides* L.)—increase of 4.63 (*p* ≤ 0.01); and flavonoids and tannins (*T. Serpyllum* L.)—increase of 3.38 (*p* ≤ 0.05).

Changes in the transcriptional activity of avian β-defensins (AvBD) were detected. According to the results of this study, the expression of *AvBD9* was increased by 8.16 times (*p* ≤ 0.01) when inulin from *C. intubus* L. was added, by 4.67 times (*p* ≤ 0.05) when ecdysterone from *R. carthamoides* L. was added, and by 1.66 times (*p* ≤ 0.05) when the flavonoid and tannin complex from *T. serpyllum* L. was added. At the same time, in the groups fed inulin and ecdysterone, high variability was found in the expression of β-defensin 9. Cv was 91.0 and 62.1% in these groups, respectively, while in the group with thyme, Cv only amounted to 10.7%.

With a significant increase in *AvBD9* expression in the ceca of broiler chickens, the transcriptional activity of the *AvBD10* gene relative to the control either increased less significantly—in the group with inulin (*C. incubus* L.) by 2.19 (*p* ≤ 0.01)—or decreased—in the group with flavonoids and tannins (*T. serpyllum* L.) by 3 (*p* ≤ 0.001). It should be noted that the *AvBD10* expression in the group with inulin was significantly higher than in the other experimental groups (*p* ≤ 0.05).

### 3.3. Morphological Parameters of Ceca

None of the ceca of the studied specimens exhibited abnormalities in their structure and development. They had typical structures for the relevant section of the digestive tube. The mucous membrane was covered with single-layer epithelium cells with underlying lamina propria.

**Figure 2:**
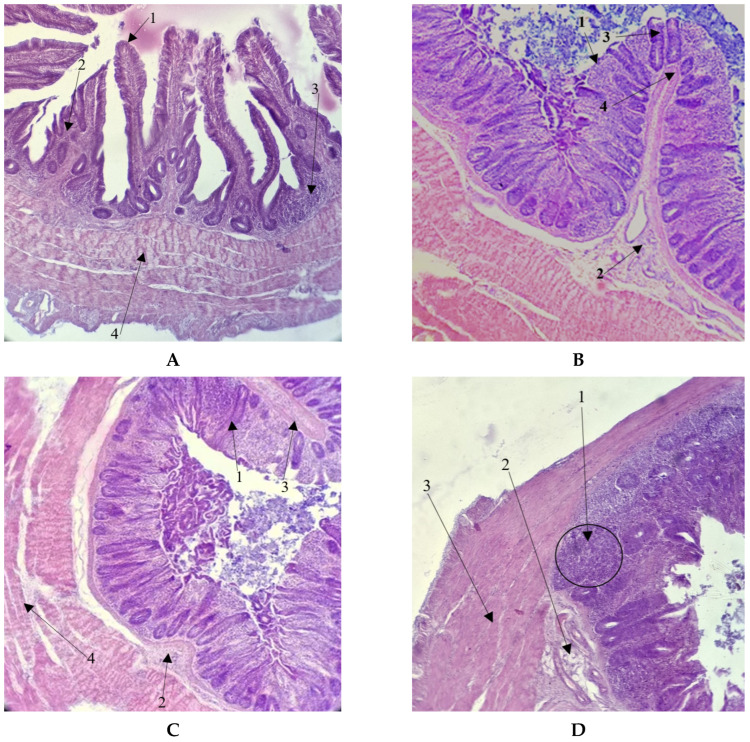
Histological structure of the cecum in broiler chickens (magnification 40×). (**A**) Histological structure of the cecum in a chicken from the CON group: 1—mucosal epithelium; 2—submucosa; 3—lymphoid clusters in submucosa; 4—muscular layer. (**B**) Mucosa within the cecum wall in a chicken from the INUL group: 1—mucosal epithelium; 2—submucosa; 3—intestinal crypts; 4—muscularis mucosae. (**C**) Histological structure of the cecum wall in a chicken from the INUL group: 1—intestinal crypts; 2—submucosa; 3—muscularis mucosae; 4—muscular layer. (**D**) Histological structure of the cecum wall in a chicken from the FLAV + TANN group: 1—clusters of lymphoid tissue; 2—submucosa; 3—muscular layer.

In the birds from all studied groups, the epithelial cells were well-preserved. This study revealed no significant destruction or degradation of the mucous membranes during preparation. The elements within lamina of the mucous membrane and the muscular layer exhibited uniform development in all chickens. The submucosa was evenly developed in all studied specimens, with the exception of birds from the FLAV + TANN group. The formation of large clusters of lymphocytes in the connective tissue of the submucosa was characteristic of all studied birds in this latter group. It should be noted that diffusely located lymphocytes were found in the organ walls of all studied birds, but Peyer’s patches-like accumulations were found only in chickens that were fed *T. serpyllum* L. extract. The histological features are shown in Figure 2.

Quantitative features characterizing the histological structures of the ceca are presented in Table 4.

It was found that the use of biologically active components in the diet of broiler chickens in all experimental groups was accompanied by a significant decrease of 67.6–48.2% (*p* ≤ 0.001) in the epithelium height of the cecum mucous membrane. At the same time, significant differences were also revealed between the experimental groups. Thus, a maximum epithelium height of 20.92 μm was recorded in the ECDS group, while the lowest was detected in the FLAV + TANN group—13.08 μm (*p* ≤ 0.001). The closest values were observed in the INUL and FLAV groups.

A reduction in the epithelium height of the ceca in chickens of the experimental groups also showed a decrease in the depth of crypts compared to those of the control group. In the FLAV + TANN group, this parameter, as well as the epithelium height, had the lowest value—206.62 μm—which was 53.4% lower (*p* ≤ 0.001) than in the group with the basic diet. Although the ECDS group had the maximum epithelium height compared to the other experimental groups, the depths of crypts in the intestinal mucosa of chickens in this group, similarly to the FLAV + TANN group (*p* = 0.246), were the lowest, namely 217.98 µm, which was also 50.9% lower (*p* ≤ 0.001) than those of the control group. In the INUL and FLAV groups, this parameter also significantly differed from the control value by 38.4 and 32.3%, respectively (*p* ≤ 0.001). The difference between these groups was reliable (*p* ≤ 0.001), but there was a dependent correspondence between the crypt depth and epithelium height in their representatives, in contrast to the ECDS and FLAV + TANN groups.

Considering the histological structure of the cecum mucous membrane in broiler chickens, changes in the thickness of muscularis mucosae of chickens in the experimental groups in comparison with broilers of the control group should be noted. Thus, when inulin from *C. intubus* L. and flavonoids from *H. perforatum* L. were used in the diets of chickens, the thickness of the muscularis mucosae increased by 6.6–7.1% (*p* ≤ 0.05), respectively; when using ecdysterone from *R. carthamoides* L., it decreased by 29.2% (*p* ≤ 0.001); and when using the complex of flavonoids and tannins from *T. serpyllum* L., it did not change. In addition, in all the experimental groups, the value of this parameter was the same, except for the ECDS group, in which the thickness of the muscularis mucosae was small.

The thickness of submucosa was reduced when using *C. intubus* L. inulin by 14.4% (*p* ≤ 0.05) compared to the control group and was significantly increased by 2.4 (*p* ≤ 0.001) when using the flavonoid and tannin complex from *T. serpyllum* L. The values of these groups differed from the other experimental groups.

The thickness of the muscular layer in the cecum changed similarly to the thickness of the muscularis mucosae. Compared to the control group, this parameter increased in the INUL and FLAV groups by 2.0–1.5 (*p* ≤ 0.001). In the ECDS group, it decreased by 1.9 (*p* ≤ 0.001), and in the FLAV + TANN group, it did not change.

### 3.4. Growth and Meat Productivity of Broiler Chickens

Based on the results of broiler chicken rearing, the effect of studied phytocomponents on bird growth, livability, and efficiency of feed utilization was established (Table 5).

The results of final weighing affirmed the positive effects of the phytocomponents used in the experiment: the live weights of broiler chickens at 35 days of age in the experimental groups were higher than in the control group by 4.1–7.5%. However, no significant difference was found. The calculated values also indicated the advantages of feeding the chickens diets with biologically active components over basic diets. In particular, while the average daily gain with a basic diet was 57.7 g, the use of extracts increased the growth rate of the birds to 60.1–62.1 g (by 4.2–7.6%).

In the absence of biologically active components that have a positive effect on the immunity and growth rate of poultry, i.e., in the basic diet, the lowest livability at 91.7% was observed in the control group, while in the experimental groups, chickens were more viable. The use of *C. intubus* L. and *H. perforatum* L. extracts prevented mortality and the use of *R. carthamoides* L. and *T. serpyllum* L. extracts minimized mortality (97.2% survival rate).

The results of the experiment showed an increase in feed consumption, which was reflected in the conversion rate. Thus, the value of this parameter in the control group was 1.58 kg, while in the experimental groups, feed costs were higher by 1.3–5.1%.

It should also be noted that despite the increase in feed costs in the experimental groups, the integral index characterizing the efficiency of broiler chicken rearing (European Production Efficiency Factor) in all experimental groups was higher than in the control group. Thus, in the control group, the index was 341.9 units, followed by the group fed with *T. serpyllum* L. extract (359.2 units) and the group fed with *H. perforatum* L. (373.0 units). The highest indices were also established in the groups fed with *R. carthamoides* L. extracts (384.7 units) and *C. intubus* L. extracts (386.4 units).

Meat productivity parameter results determined through the anatomical cutting of the birds are presented in Table 6.

In terms of gutted carcass weight, there were no significant differences between the groups, but the relative slaughter yield parameter showed an advantage for the male birds of the group fed with *T. serpyllum* L. extract, with the index in this group being higher than in the control group by 6.1 abs.%.

Better development of breast muscles was found in the male chickens of the experimental groups compared to the chickens of the control group. Thus, compared to the control group, breast muscle mass was 16.9% higher in the INUL group, 18.1% higher in the ECDS group, and 23.1% higher in the FLAV + TANN group (*p* ≤ 0.05). Breast muscle yield was higher in the FLAV + TANN group by 3.9 abs.% (*p* ≤ 0.05).

The highest fat content was found in the carcasses of chickens fed with *H. perforatum* L. extract. The weight and fat yield in female birds in this group, and therefore in the group as a whole, were twice as high as those in the control group (*p* ≤ 0.05).

## 4. Discussion

### 4.1. Expression of Immunity Genes in the Ceca of Broiler Chickens

One of the key objectives of phytobiotics in the nutrition of monogastric animals is to increase their productivity by stimulating organism resistance. A number of scientific studies have demonstrated that this can be primarily achieved by improving intestinal health [7,9].

The ceca are important elements of the large intestines of poultry, the functions of which include the synthesis of immunoglobulins and antibodies; the creation of favorable conditions for the development of normal microbiota through the suppression of pathogenic and opportunistic microorganisms; water utilization and absorption; and the transformation of uric acid into amino acids, as well as the digestion and absorption of nutrients [23], especially the participation in the breakdown and digestion of fiber [24]. This organ is of great interest regarding the assessment of the transcriptional activity of immunity genes, since ceca are colonized by microorganisms that influence poultry resistance and productivity and 70% of the tissues and organs of the immune system are found near the intestine or within it [25].

The *IL8L2* gene/protein is one of the key pro-inflammatory cytokines and plays a central role in the initiation of inflammatory reactions against bacterial and viral infections [26]. Taking into account the absence of pathogenic factors and identical feed and housing conditions for all groups, the cytokine expression increase in the cecum tissues of chickens can be considered the result of the positive effects of the biologically active components on intestinal barrier function. Intestinal microbiota are known to play a key role in the activation of the immune defense of an organism, and this is ensured by, among other things, the modulation of inflammatory reactions through the production of cytokines [27]. Since chicory inulin is a prebiotic and flavonoids and tannins in thyme have antibacterial properties, the use of these extracts may have promoted the better development of normal flora, which, in turn, was reflected in the activity of innate immunity. Similar results established by Laptev et al. showed a five-time increase in *IL8L2* expression in intestinal tissues when a mixture of essential oils of eucalyptus (*Eucalyptus obliqua* L’Her), garlic (*Állium satívum* L.), lemon (*Citrus limon* L.), and thyme (*Thymus serpyllum* L.) was used in the diet of healthy broiler chickens [20]. The increase in interleukin transcriptional activity in the group of chickens fed with *R. carthamoides* L. extract standardized to plant steroids was associated with the intensification of protein metabolism, which was confirmed by the results of the rearing and anatomical cutting of the birds (Table 5 and Table 6). Sugihara et al. reported that amino acids play a significant role in the metabolic reactions of the intestinal microbiota and indicate a reduction in cytokine synthesis when they are deficient [28]. The increase in protein metabolism under the influence of ecdysterone may have contributed to the more active use of amino acids by the intestinal microbiota, which also had a positive effect on innate intestinal immunity.

Avian β-defensins *(AvBD)* are genes encoding antimicrobial peptides with a broad spectrum of activity against bacteria, fungi, and viruses. Together with cytokines and Toll-like receptors, they are the key marker genes of innate immunity [29,30]. The functions of avian β-defensins include antibacterial activity, chemotaxis, the activation of immune cells, the modulation of inflammation and autophagy, and the maintenance of epithelial homeostasis through the activation of mucin synthesis [31]. Thus, *AvBD* family genes are an important component of immunity in poultry. The increased expression of *AvBD9* under the influence of *C. intubus* L., *R. carthamoides* L., and *T. serpyllum* L. indicates the immunomodulatory properties of these phytobiotics; however, when introducing inulin and ecdysterone into the diets of chickens, the high level of trait variability may be associated with the indirect action of these biologically active components through the stabilization of intestinal microbiota and the intensification of protein metabolism, as in the case with the change in the expression of interleukin-8. In contrast, flavonoids and tannins from *T. serpyllum* L. had a direct effect on the immune defense of the intestinal mucosa, which was confirmed by the high homogeneity of *AvBD9* transcriptional activity in the chickens of the respective group. At the same time, the absence of a significant effect in the group fed with *H. perforatum* L. extract, which contains a high concentration of flavonoids, may indicate that the increased expression of antimicrobial peptide genes in the group fed with *T. serpyllum* L. is primarily due to the effect of tannins. The positive effect of tannins on the composition of microorganisms and intestinal health has been established in a number of studies [32,33,34].

Taking into account that the expression of *AvBD9* and *AvBD10* genes in all groups had the same pattern of change relative to the control, it can be assumed that relatively low expression of β-defensin 10 in the experimental groups was compensated for by the high expression of β-defensin 9. Thus, this pattern is caused by the interdependent mechanisms of expression between the studied peptides, and the decrease in AvBD10 expression compared to the control group did not negate the positive effect of the analyzed phytochemical compounds on intestinal immunity modulation. In the above-mentioned study by Laptev et al. devoted to the effect of complex phytobiotic on the expression of immunity genes, a similar pattern was established, but in a different direction. With a significant increase in the expression of *AvBD10*, the expression of *AvBD9* was considerably lower both when the preparation was used on intact birds throughout the experiment and on birds infected with *Salmonella enteritidis* three weeks after the introduction of infection [20]. A similar pattern in the expression of β-defensins in poultry under the influence of various paratypic factors can be observed in a number of studies [35,36].

The results of the *IRF7* gene expression analysis indicated that there were no significant differences between the control and experimental groups, which indicates the absence of a pronounced modulation of immunity against viruses by biologically active components of the studied extracts. Also notable was the absence of identified immunomodulatory properties in flavonoids of *H. perforatum* L., both in terms of the activation of pro-inflammatory cytokine synthesis and the antimicrobial defense of the intestine.

Thus, biologically active compounds of the experimental extracts (inulin in *C. intubus* L. extract, ecdysterone in *R. carthamoides* L. extract, and tannins in *T. serpyllum* L. extract) have immunomodulatory properties, which are expressed as the activation of interleukin-8 and β-defensins synthesis 9–10.

### 4.2. Morphological Parameters of Ceca

In addition to molecular methods, gastrointestinal markers should be used to assess intestinal health [37]. Homeostasis in the intestine is maintained by immune mechanisms occurring in the wall, including the development of large lymphoid nodules in the mucous membrane, which provide important defense reactions [23]. The related Peyer’s patches play an important role in animal immunity, in particular, in the capture and disposal of pathogens, in antigen presentation, and in the activation of defense reactions in the mucous membrane of the gastrointestinal tract, involving dendritic cells and macrophages, T- and B-cells, and immunoglobulin A [38]. According to some reports, Peyer’s patches are a key target for oral immunostimulants [39]. Thus, the appearance of large clusters of lymphoid follicles similar to Peyer’s patches in the ceca walls of broiler chickens in the FLAV + TANN group should be considered evidence of an increase in the immune function of the intestine under the influence of flavonoids and tannins in *T. serpyllum* L. The immunomodulatory properties of satsuma mandarin (*Citrus unshiu*) zest extract have been established previously, and it contains flavonoids, which were expressed in the stimulation of Peyer’s patches formation in the intestines of mice [40].

The key role of the intestinal ceca in the poultry metabolism is the enzymatic processing of feed fiber by microorganisms; fermentation products such as various gasses; lactic acid; and short-chain fatty acids used in the further metabolism of the host organism [41]. Taking into account that in the control group, the structure of epithelial cells was not changed, this indicates that the absence of inflammatory and apoptotic processes could cause destructive cell growth; for instance, the lowest epithelial height, and therefore the lowest absorptive surface, in the experimental groups could be associated with a decrease in the efficiency of cellulose hydrolysis product assimilation. This can be indirectly confirmed by feed costs per 1 kg of gain, which were higher in the experimental groups compared to the control group. However, to better understand the effects of the studied plant extracts on the processes associated with the biodegradation of non-starch polysaccharides, it is necessary to further study the digestibility of crude fiber and the development of cellulosolytic microorganisms in the ceca of broiler chickens, as well as to conduct morpho-histological evaluations of the small intestine, which plays the most important role in the digestion and assimilation of feed nutrients. The reduction in the absorptive surface when using phytobiotics was established previously. When used in chicken diets, the combination of the aqueous and cyclodextrin plant extracts of *Origanum vulgare* subsp. *hirtum* L., *Alliumsativum* L., *Crithmum maritimum* L., and *Camelina sativa* L. Crantz leads to lower villi in the ileum, while cyclodextrin alone reduces the height of villi in the duodenum of broiler chickens [42].

At the same time, a reduction in the height of the epithelium was accompanied by a decrease in the depth of crypts, which, contrarily, indicates an increase in the efficiency of digestive processes [43]. A decrease in crypt depth in the small intestine may result in increased enzymatic activity and absorption capacity [44]. This could be applied to the cecum in terms of the absorption of the metabolites of cellulosolitic microorganisms. However, crypt depth was of primary interest in this study as a morpho-histological indicator characterizing the protective function of the poultry intestine. Deeper crypts in the intestinal mucosa are known to indicate more intensive tissue regeneration due to the presence of pathogenic microorganisms and their toxins in the walls [45]. In this study, the lowest depth of crypts was observed in the FLAV + TANN group, which, combined with the accumulation of lymphoid clusters in the connective tissue of the submucosal layer and the extremely low variability in the expression of genes related to antimicrobial activity, clearly indicates the immunomodulatory and protective effects of flavonoids and tannins from the extract of *T. serpyllum* L. on the intestinal tract. The lowest crypt depth in the INUL and ECDS groups was also accompanied by a significant increase in the expression of genes associated with resistance, indicating the positive effects of the extracts on these groups. In contrast, the FLAV group showed the greatest crypt depth compared to the other experimental groups, and the expression of immune-related genes changed insignificantly compared to the control group. Thus, the use of the *H. perforatum* L. extract does not considerably improve the condition of the cecum in poultry. The positive effect of phytobiotics on large intestine health has been established previously, including in infected poultry, in particular, where a reduction in crypt depth in ceca was reported when using lemon peel powder (*Citrus limon* L.) in the diet of broiler chickens infected with *E. tenella* [46]. In addition, numerous studies indicate a reduction in crypt depth in the small intestine through the use of various phytobiotics, namely puncturevine powder (*Tribulus terrestris* L.) [47], brown algae extract (*Ascophyllum nodosum* L.) [48], and cinnamon oil (*Cinnamomum verum* J.Presl) [49].

The muscularis mucosae of the mucous membrane and the muscular layer determine the speed and strength of the intestinal peristalsis, and thus the nature of chyme movement and the duration of its retention in the cecal appendices [44]. The maximum thickness of these elements was found in the INUL and FLAV groups, while the minimum was observed in the ECDS group. Inulin and flavonoids are known to alter the microbiomes of the cecal appendices in poultry [50,51,52] and are used to correct the microbial community of the gastrointestinal tract, while ecdysterone is primarily aimed at enhancing protein biosynthesis. The use of *C. intubus* L. and *H. perforatum* L. extracts is likely to have contributed to the development of cellulolytic microbiota in the cecal appendices, which facilitated the more efficient breakdown of feed fiber, eliminated the need for the prolonged retention of the contents, and accounted for rapid evacuation during intense peristalsis. In the ECDS group, on the other hand, contents were retained for a longer time period due to the low cellulosolytic activity of microorganisms without the need for the development of intestinal wall elements in order to provide active peristalsis. In the FLAV + TANN group, the parameters of muscular elements did not differ from those of the control group, apparently because the substances of *T. serpyllum* L. directly affected the intestinal histostructure to a greater extent, which was confirmed by other histological parameters and the results of molecular genetic studies. However, this emphasizes the need for the further study of the intestinal microbial community of chickens fed with these extracts.

### 4.3. Growth and Meat Productivity of Broiler Chickens

Intestinal health is one of the key factors predetermining the level of productivity of poultry [53], which was proven by the results of the present study. The best results in terms of the growth of broiler chickens were observed in the experimental group fed *R. carthamoides* L. extract, which is apparently due to the intensification of skeletal muscle growth when provided with the phytosteroid ecdysterone. The positive effect of the use of phytosteroid compounds in the diet of broiler chickens was shown in the work of Naji et al., who demonstrated that the live weight of chickens at the age of 42 days increased by 3.1–14.1% relative to the control group when phytosteroids castasterone and β-sitosterol were added to their diets at different input levels [54].

Considering the patterns of changes in feed conversion in poultry when using phytobiotics in their diets, it should be noted that, in their review of the global scientific literature, Baghno et al. observed that phytobiotics, unlike probiotics, not only serve as stabilizers of intestinal microbiota but also have an impact on digestive processes by stimulating the secretion of endogenous enzymes and improving the palatability of the feed, leading to increased consumption [55]. The results of our study confirmed this result. The phenolic compounds in the extracts of *H. perforatum* L. and *T. serpyllum* L. are likely to have enhanced the palatability of the feed, stimulating its consumption by the chickens. The relatively low values in the groups fed with inulin from *C. intubus* L. (1.63 kg) and phytosterol from *R. carthamoides* L. (1.60 kg) should also be noted. Thus, the chickens in these groups were characterized not only by the highest average daily gain but also by the lowest feed costs, indicating the advantage of these extracts over others.

Regarding the slaughter yield of chickens fed with *T. serpyllum* L. extract, practically identical results were obtained in the study by Kishnyaykina et al., where the slaughter yield in the control group was 69% (in our case—68.7%); the use of this extract at 10 mg/kg of live weight increased the value of the indicator by 5.5% to 74.5% (in our case—74.8%) [56]. The similarity of the results testifies to the reliability of the obtained data and the positive effect of the phenolic compounds of *T. serpyllum* L. extract on the slaughter quality of poultry.

It should be noted that, in the group of chickens fed with *H. perforatum* L. extract standardized to flavonoids, there was no significant increase in the expression of immunity genes and slaughter quality. At the same time, the highest meat productivity was observed in chickens fed *T. serpyllum* L. extract, which had the highest homogeneity with respect to the transcriptional activity of resistance genes. This suggests the dependence of the slaughter qualities of poultry on the level of immunity activity.

In addition, it should be mentioned that significant differences in slaughter quality were found only in male birds, which is probably due to sexual dimorphism and a more pronounced biological response, manifested in the growth of skeletal muscles, in response to the action of biologically active components in their diet [57]. For the same reason, significant differences in carcass fat content were observed in females, which are more prone to fat tissue accumulation.

## 5. Conclusions

Thus, the inclusion of biologically active components extracted from *C. intubus* L. (inulin), *R. carthamoides* L. (ecdisterone), and *T. serpyllum* L. (tannins and flavonoids) in broiler chicken diets contributes to the activation of innate immunity by stimulating the production of cytokines and antimicrobial peptides and improving the morphofunctional features of ceca, which has a positive effect on the safety, growth, and meat productivity of poultry. The extract of *H. perforatum* L., standardized to flavonoids, had no positive effect on immunity or the slaughter quality of poultry and significantly increased the content of fatty tissue in the carcass. The results of the study indicate the possibility of replacing feed antibiotics in poultry rearing with natural growth stimulants and could be the basis for the further development of new phytocombinations, which could modulate immunity, ensure intestinal health, and increase the productivity of broiler chickens as part of environmentally friendly poultry production.

## Figures and Tables

**Table 1:** Experimental groups.

Groups	Number of Animals in Each Group (*n*)	Broiler Chicken Feeding Program
CON	36	Basic diet (BD) for broiler chickens: Starter, Grower, and Finisher feeds without phytochemicals.
INUL	36	BD + INUL: 405.0 g/t in Starter/Grower feeds, 504.0 g/t in Finisher feed
FLAV	36	BD + FLAV: 22.6 g/t in Starter/Grower feeds, 31.4 g/t in Finisher feed
ECDS	36	BD + ECDS: 0.9 g/t in Starter/Grower feeds, 1.1 g/t in Finisher feed
FLAV + TANN	36	BD + FLAV: 36.8 g/t in Starter/Grower feeds, 46.0 g/t in Finisher feed + TANN 7.2 g/t in Starter/Grower feeds, 9.0 g/t Finisher feed

CON—control; INUL—inulin in common chicory (*C. intubus* L.); FLAV—flavonoids in St. John’s wort (*H. perforatum* L.); ECDS—ecdysterone in maral root (*R. carthamoides* L.); FLAV + TANN—flavonoids and tannins in creeping thyme (*T. serpyllum* L.).

**Table 2:** Nutritional value of compound feeds for broiler chickens.

Nutrients	Age of Poultry (Days)		
	0–10 (Starter)	11–22 (Grower)	23–35 (Finisher)
	Nutritional Value (%)		
Metabolic energy (ME) (kcal/100 g)	305.00	299.00	303.00
Crude protein	23.00	21.50	18.50
Assimilable lysine	1.44	1.33	1.20
Assimilable methionine	0.67	0.67	0.63
Assimilable methionine + cystine	1.04	1.03	0.94
Assimilable threonine	0.97	0.91	0.81
Assimilable tryptophan	0.26	0.22	0.21
Crude fiber	3.69	4.75	5.00
Essential extract	3.73	5.38	5.50
Linoleic acid	1.74	2.63	2.81
Calcium	1.10	0.87	0.78
Total phosphorus	0.69	0.67	0.61
Assimilable phosphorus	0.58	0.42	0.51
Sodium	0.17	0.17	0.18
Chlorine	0.17	0.19	0.20
Vitamin B4 (mg/kg)	1568.00	1568.00	1540.00

**Table 3:** Nucleotide sequences of primers.

Gene	Primers	Author
*ACTB* (β-actin)	F: CTGTGCCCATCTATGAAGGCTA R: ATTTCTCTCTCGGCTGTGGTG	Laptev G.Yu. et al., 2023 [22]
*IL8L2* (interleukin 8-like 2)	F: GGAAGAGAGGTGTGCTTGGA R: TAACATGAGGCACCGATGTG	
*AvBD9* (β-defensin 9)	F: AACACCGTCAGGCATCTTCACA R: CGTCTTCTTGGCTGTAAGCTGGA	
*AvBD10* (β-defensin 10)	F: GCTCTTCGCTGTTCTCCTCT R: CCAGAGATGGTGAAGGTG	
*IRF7* (interferon regulatory factor 7)	F: ATCCCTTGGAAGCACAACGCC R: CTGAGGCAACCGCGTAGACCTT	

**Table 4:** Parameters of the histological structure of the ceca (μm).

Parameters	Group					*p*-Value
	CON	INUL	FLAV	ECDS	FLAV + TANN	
Epithelium height	40.42 ± 0.360 ^a^	15.64 ± 0.396 ^b^	16.15 ± 0.445 ^b^	20.92 ± 0.395 ^c^	13.08 ± 0.193 ^d^	0.000
Crypt depth	443.58 ± 5.931 ^a^	273.38 ± 3.754 ^b^	300.39 ± 4.238 ^c^	217.98 ± 2.103 ^d^	206.62 ± 2.342 ^d^	0.000
Thickness of muscularis mucosae	17.51 ± 0.231 ^a^	18.66 ± 0.193 ^b^	18.75 ± 0.383 ^bc^	12.40 ± 0.241 ^d^	17.87 ± 0.323 ^ab^	0.000
Thickness of submucosa	47.64 ± 0.818 ^a^	40.80 ± 0.465 ^b^	51.11 ± 0.998 ^a^	47.75 ± 1.005 ^a^	113.29 ± 3.410 ^c^	0.000
Thickness of muscular layer	283.61 ± 3.377 ^a^	579.87 ± 5.567 ^b^	430.99 ± 4.819 ^c^	151.59 ± 2.845 ^d^	291.59 ± 3.032 ^a^	0.000

Values are expressed as means ± standard error. Means denoted within the same row with different superscripts are significant (*p* ≤ 0.05).

**Table 5:** Zootechnical parameters of broiler chicken rearing.

Parameters	Group					*p*-Value
	CON	INUL	FLAV	ECDS	FLAV + TANN	
Initial number of birds	36	36	36	36	36	-
Live weight at 35 days, g *	2061.9 ± 51.62	2204.6 ± 50.38	2167.3 ± 55.63	2216.1 ± 48.91	2146.8 ± 44.50	0.233
Average daily gain, g	57.7	61.8	60.7	62.1	60.1	-
Livability of birds, %	91.7	100.0	100.0	97.2	97.2	-
Feed consumption per 1 kg of gain, kg	1.58	1.63	1.66	1.60	1.66	-
EPEF, units	341.9	386.4	373.0	384.7	359.2	-

* Values are expressed as means ± standard error.

**Table 6:** Meat productivity parameters of broiler chickens.

Parameters	Groups					*p*-Value
	CON	INUL	FLAV	ECDS	FLAV + TANN	
Total sample						
Number of individuals	6	6	6	6	6	-
Weight of gutted carcass, g	1393.9 ± 50.05	1513.4 ± 61.78	1499.7 ± 43.10	1524.6 ± 55.71	1532.3 ± 66.63	0.417
Slaughter yield, %	70.2 ± 1.60	73.0 ± 0.65	72.3 ± 1.04	73.2 ± 1.33	73.4 ± 0.82	0.291
Weight of breast muscles, g	440.8 ± 5.43	482.7 ± 19.87	463.4 ± 21.36	491.8 ± 17.20	495.4 ± 26.39	0.270
Breast muscle yield, %	22.3 ± 0.88	23.3 ± 0.43	22.3 ± 0.45	23.6 ± 0.31	23.7 ± 0.52	0.208
Fat weight, g	25.4 ± 1.59 ^a^	38.9 ± 4.54 ^ab^	52.7 ± 5.44 ^b^	36.3 ± 2.88 ^a^	32.8 ± 2.52 ^a^	0.001
Fat yield, %	1.3 ± 0.12 ^a^	1.9 ± 0.18 ^ab^	2.6 ± 0.31 ^b^	1.7 ± 0.10 ^a^	1.6 ± 0.16 ^a^	0.001
♂						
Number of individuals	3	3	3	3	3	-
Weight of gutted carcass, g	1491.2 ± 31.14	1645.3 ± 40.69	1586.8 ± 35.51	1628.8 ± 54.92	1674.9 ± 33.71	0.063
Slaughter yield, %	68.7 ± 1.24 ^a^	72.4 ± 1.34 ^ab^	70.3 ± 0.97 ^ab^	72.3 ± 1.75 ^ab^	74.8 ± 0.71 ^b^	0.052
Weight of breast muscles, g	446.3 ± 7.79 ^a^	521.8 ± 18.34 ^bc^	502.2 ± 17.71 ^ac^	527.0 ± 13.82 ^bc^	549.6 ± 18.54 ^bc^	0.009
Breast muscle yield, %	20.6 ± 0.59 ^a^	23.0 ± 0.70 ^ab^	22.2 ± 0.56 ^ab^	23.4 ± 0.45 ^ab^	24.5 ± 0.66 ^b^	0.052
Fat weight, g	23.0 ± 0.85	44.5 ± 7.95	49.1 ± 8.41	41.7 ± 1.61	29.8 ± 4.57	0.042
Fat yield, %	1.1 ± 0.05	2.0 ± 0.36	2.2 ± 0.36	1.9 ± 0.08	1.3 ± 0.22	0.054
♀						
Number of individuals	3	3	3	3	3	-
Weight of gutted carcass, g	1296.7 ± 45.81	1381.5 ± 4.02	1412.6 ± 21.12	1420.4 ± 40.57	1389.6 ± 26.51	0.112
Slaughter yield, %	71.7 ± 3.00	73.5 ± 0.21	74.3 ± 0.73	74.2 ± 2.21	72.1 ± 0.95	0.767
Weight of breast muscles, g	435.2 ± 7.49	443.7 ± 10.50	424.6 ± 21.53	456.5 ± 6.77	441.3 ± 14.41	0.566
Breast muscle yield, %	24.1 ± 0.71	23.6 ± 0.58	22.3 ± 0.82	23.8 ± 0.48	22.9 ± 0.47	0.312
Fat weight, g	27.9 ± 2.43 ^a^	33.3 ± 2.93 ^a^	56.2 ± 8.04 ^b^	31.0 ± 3.16 ^a^	35.8 ± 1.27 ^a^	0.006
Fat yield, %	1.5 ± 0.12 ^a^	1.8 ± 0.15 ^a^	3.0 ± 0.46 ^b^	1.6 ± 0.19 ^a^	1.9 ± 0.09 ^ab^	0.011

Values are expressed as means ± standard error. Means denoted within the same row with different superscripts are significant (*p* ≤ 0.05). ♂—males; ♀—females.

## Data Availability

The raw data supporting the conclusions of this article will be made available by the authors without undue reservations. The data presented in this study are available upon request from the corresponding author.
